# Depressive and anxiety symptoms after the 2024 flooding in Rio Grande do Sul, Brazil: findings of the PAMPA cohort

**DOI:** 10.1590/0102-311XEN096225

**Published:** 2026-02-16

**Authors:** Natan Feter, Natália Schröeder, Felipe Delpino, Luísa da Silva, Eduardo Caputo, Isabel Paz, Juliana Rocha, Jayne Feter, Yohana Vieira, Marcelo da Silva, Felipe Reichert, Airton Rombaldi

**Affiliations:** 1 Department of Biological Sciences, University of Southern California, Los Angeles, U.S.A.; 2 Universidade Federal de Pelotas, Pelotas, Brasil.; 3 Faculdade de Medicina, Universidade Federal do Rio Grande do Sul, Porto Alegre, Brasil.; 4 Programa de Pós-graduação em Enfermagem, Universidade Federal de Pelotas, Pelotas, Brasil.; 5 Faculdade de Medicina, Universidade Federal de Pelotas, Pelotas, Brasil.; 6 School of Public Health, Brown University, Providence, U.S.A.; 7 Programa de Pós-graduação em Ciências do Movimento Humano, Universidade Federal do Rio Grande do Sul, Porto Alegre, Brasil.; 8 Faculdade de Medicina, Universidade Federal do Rio Grande, Rio Grande, Brasil.; 9 Programa de Pós-graduação em Enfermagem, Universidade Federal de Pelotas, Pelotas, Brasil.

**Keywords:** Climate Change, Depression, Anxiety, Floods, Mudança Climática, Depressão, Ansiedade, Inundações, Cambio Climático, Depresión, Ansiedad, Inundaciones

## Abstract

The 2024 flooding in Rio Grande do Sul, Brazil, was the worst natural disaster in the history of the state. We aimed to examine the impact of flooding on individuals’ lives and depressive and anxiety symptoms among residents of Rio Grande do Sul. We analyzed data from the *Prospective Study on Mental and Physical Health in Adults* (PAMPA), conducted with adults from Rio Grande do Sul. Participants completed an online questionnaire between September and November 2024. Depressive and anxiety symptoms were assessed using the *Hospital Anxiety and Depression Scale*, in which scores above eight indicated moderate-to-severe symptoms. The flooding impact was measured via 12 questions on direct and indirect consequences (e.g., energy shortages, displacement, material losses) and categorized into tertiles: low, medium, and high burden. Among the 2,494 participants (mean age: 43.1 ± 15.1 years; 69.6% women), 83.8% reported being affected by the flooding, and 29.7% experienced a high burden. Displaced participants were more likely to report anxiety (PR = 1.24; 95%CI: 1.09, 1.41) and depressive symptoms (PR = 1.32; 95%CI: 1.10, 1.58). High burden was associated with anxiety (PR = 1.72; 95%CI: 1.49, 1.97) and depressive symptoms (PR = 1.52; 95%CI: 1.25, 1.86). Moderate-to-severe anxiety and depressive symptoms were 77% and 129%, respectively, higher than expected. The 2024 flooding had a profound impact on mental health, in which individuals who experienced a greater burden had higher rates of moderate-to-severe symptoms of anxiety and depression.

## Abstract (pt)

A enchente de 2024 no Rio Grande do Sul, Brasil, foi o pior desastre natural da história do estado. Nosso objetivo foi examinar o impacto das enchentes na vida dos indivíduos e os sintomas depressivos e ansiosos entre os residentes do Rio Grande do Sul. Analisamos dados do *Estudo Prospectivo sobre Saúde Mental e Física em Adultos* (PAMPA), realizado com adultos do Rio Grande do Sul. Os participantes responderam a um questionário online entre setembro e novembro de 2024. Os sintomas depressivos e ansiosos foram avaliados usando a *Escala Hospitalar de Ansiedade e Depressão*, com pontuações acima de oito indicando sintomas moderados a graves. O impacto das enchentes foi medido por meio de 12 perguntas sobre consequências diretas e indiretas (p.ex.: escassez de energia, deslocamento, perdas materiais) e categorizadas em tercis: baixa, média e alta carga. Entre os 2.494 participantes (idade média: 43,1 ± 15,1 anos; 69,6% mulheres), 83,8% relataram ter sido afetados pelas enchentes e 29,7% sofreram uma grande sobrecarga. Os participantes deslocados foram mais propensos a relatar sintomas de ansiedade (RP = 1,24; IC95%: 1,09, 1,41) e sintomas depressivos (RP = 1,32; IC95%: 1,10, 1,58). Alta sobrecarga foi associada a sintomas de ansiedade (RP = 1,72; IC95%: 1,49, 1,97) e sintomas depressivos (RP = 1,52; IC95%: 1,25, 1,86). Sintomas de ansiedade e depressão moderados a graves foram 77% e 129%, respectivamente, maiores do que a prevalência esperada. A enchente de 2024 teve um impacto profundo na saúde mental, com indivíduos que experimentaram uma sobrecarga maior exibindo taxas mais altas de sintomas moderados a graves de ansiedade e depressão.

## Abstract (es)

Las inundaciones de 2024 en Rio Grande do Sul, Brasil, fueron el peor desastre natural en la historia del estado. Nuestro objetivo era examinar el impacto de las inundaciones en la vida de las personas y los síntomas de depresión y ansiedad entre los residentes de Rio Grande do Sul. Analizamos los datos del *Estudio Prospectivo sobre Salud Mental y Física en Adultos* (PAMPA), realizado con adultos de Rio Grande do Sul. Los participantes completaron un cuestionario en línea entre septiembre y noviembre de 2024. Los síntomas depresivos y de ansiedad se evaluaron utilizando la *Escala de Ansiedad y Depresión Hospitalaria*, en la que las puntuaciones superiores a ocho indicaban síntomas de moderados a graves. El impacto de las inundaciones se midió mediante 12 preguntas sobre las consecuencias directas e indirectas (por ejemplo, escasez de energía, desplazamientos, pérdidas materiales) y se clasificó en terciles: afectación baja, media y alta. De los 2.494 participantes (edad media: 43,1 ± 15,1 años; 69,6% mujeres), el 83,8% declaró haberse visto afectado por las inundaciones y el 29,7% experimentó un alto grado de afectación. Los participantes que se vieron desplazados eran más propensos a declarar síntomas de ansiedad (RP = 1,24; IC95%: 1,09, 1,41) y síntomas depresivos (RP = 1,32; IC95%: 1,10, 1,58). Una mayor afectación se asoció con síntomas de ansiedad (RP = 1,72; IC95%: 1,49, 1,97) y con síntomas depresivos (RP = 1,52; IC95%: 1,25, 1,86). La prevalencia de síntomas moderados-graves de ansiedad y depresión fue un 77% y un 129% superior, respectivamente, a la prevalencia esperada. Las inundaciones de 2024 tuvieron un impacto profundo en la salud mental, y las personas que sufrieron una mayor afectación presentaron tasas más elevadas de síntomas moderados-graves de ansiedad y depresión.

## Introduction

Floods are the most common climate-related natural disaster and some projection indicate an increase in their frequency, severity, and duration due to ongoing climatic changes ^1^ ^,^ ^2^. Climate change plays a significant role in intensifying and increasing the frequency of floods in certain regions. For example, rising temperatures accelerate water evaporation from land and oceans, leading to more intense and frequent precipitation ^3^ ^,^ ^4^. Additionally, urbanization, deforestation, and poor drainage worsen floods, endangering infrastructure, water supplies, and ecosystems ^5^ ^,^ ^6^.

Over the past four decades, flood-related disasters have affected a growing number of people, with an estimated 1.81 billion individuals directly exposed ^1^. Floods happen less often in South America, where research on the subject remains limited ^7^. However, an unprecedented flood devastated the Brazilian state of Rio Grande do Sul in April 2024, impacting nearly 2.4 million people (out of 11.2 million living in the state), causing 183 deaths, leaving 27 individuals missing, and affecting 478 out of 497 municipalities ^8^ ^,^ ^9^ ^,^ ^10^.

Following disasters, the prevalence of mental health disorders can differ: anxiety (2.2%-84%), depression (3.23%-52.7%), and posttraumatic stress disorder - PTSD (2.6%-52%) ^11^. Poor mental health after floods often relates to physical illness, financial loss, and social disruption ^7^, particularly in areas with weak healthcare infrastructure. Moreover, vulnerable groups, such as women, children, marginalized communities, and displaced individuals, are disproportionately affected due to social inequality and limited healthcare access ^12^.

While flood-related mental health has been studied globally, research is scarce in South America. Understanding factors such as anxiety, resilience, and mental health outcomes remains a challenge ^13^. Evidence is critical to guide disaster response and mental health interventions. Thus, this study aimed to assess the impact of flooding on lives and depressive and anxiety symptoms among adults in Rio Grande do Sul. This major natural disaster offered a unique opportunity for study, although rare in the region.

## Methods

We conducted an observational study that integrated cross-sectional and longitudinal analyses using data from the *Prospective Study on Mental and Physical Health in Adults* (PAMPA) cohort, a large population-based study conducted in the state of Rio Grande do Sul, the southernmost state in Brazil ^14^ ^,^ ^15^. Participants were recruited via multiple strategies, including advertisements on university websites, local and regional media outlets, social media platforms, and personal networks to maximize diversity in sociodemographic characteristics and health profiles. Eligibility criteria included being an adult (≥ 18 years old) and residing in Rio Grande do Sul during each wave of survey. Individuals provided electronical informed consent prior to participation. The study protocol was approved by the institutional review board of the Physical Education Faculty at the Federal University of Pelotas, Brazil (CAAE: 31906920.7.0000.5313).

The PAMPA cohort was launched in June 2020 in response to the COVID-19 pandemic to monitor long-term consequences of the crisis on physical and mental health. Six waves of data collection have been conducted since its inception: (1) baseline in June 2020; (2) December 2020; (3) June 2021; (4) 2022; (5) 2023, and (6) September 2024. Wave 6, initially scheduled for June 2024, was postponed to September due to the severe flooding that affected Rio Grande do Sul. Each wave consisted of an online questionnaire distributed to all baseline participants who had provided contact information.

At baseline, 2,260 adults completed the questionnaire. The mean age of participants was 37.5 years (18-80), with about 76.4% women across the state. Among these, 1,326 participants (58.6%) consented to be recontacted by providing at least one type of contact information (telephone number, Facebook, or Instagram usernames). They formed the eligible follow-up cohort. Retention rates across waves were 50.1% at wave 2, 48.3% at wave 3, 34.8% at wave 4, 57.9% at wave 5, and 53.7% at wave 6.

Sociodemographic characteristics (age, gender, occupational status, race and ethnicity, marital status, and schooling) and mental health outcomes (symptoms of depression and anxiety) were assessed consistently across all study waves ^14^ ^,^ ^15^. The impact of the 2024 flooding was evaluated at wave 6 (September 2024). All study procedures were approved by the university research ethics committee, and informed consent was obtained prior to each wave of data collection.

### Flooding impact evaluation

We adapted the questionnaire created by Martin et al. ^16^ to assess the impact of the flood on individuals’ lives. The questionnaire addresses both direct and indirect consequences, such as disrupted supply of energy, food, or drinkable water; material losses; flood-related infectious diseases; pet loss; blocked roads or airports; and impaired health treatment. We used questions 18 to 29 (Supplementary Material; https://cadernos.ensp.fiocruz.br/static//arquivo/suppl-e00096225_7615.pdf). Each positive answer was scored as one, so the sum of answers created a continuous burden score ranging from 0 to 12. The answers were also categorized into tertiles to classify participants into three burden categories: low, medium, and high burden. We also asked participants whether they had to leave home due to the flooding. In case of a positive answer, they explained if their home was affected and if there were partial or total losses.

### Anxiety and depressive symptoms

We assessed depressive and anxiety symptoms using the *Hospital Anxiety and Depression Scale* (HADS) ^17^. Each domain (i.e., depression and anxiety) contains seven items, of which participants scored between 0 and 3 according to the frequency of such symptoms during the previous week. Therefore, each domain had a maximum score of 21 and participants who scored less than 7 were classified as non-symptomatic for that domain (i.e., anxiety or depression). Scores from 8 to 10 were considered as mild risk, from 11 to 14 as moderate risk, and 15 and higher as severe risk of depression and/or anxiety ^17^. We merged the two extreme categories to examine the association between flood impact and the proportion of moderate-to-severe anxiety and depressive symptoms.

### Covariates

All multivariable models included the following covariates: macroregions of the state (Serra, Norte, Missioneira, Centro-oeste, Vales, Metropolitana, and Sul), age (years), gender (woman, man), race and ethnicity (Asians, Black, Brazilian Indigenous, Mixed-race, White, other), schooling (high school or lower, undergraduate degree, graduate or higher), income (minimum wage), and occupational status (not working, working, studying, working and studying, retired, other).

### Statistical analysis

We expressed categorical variables as frequencies and percentages and described continuous variables using the mean and standard deviation. We illustrated the distribution of the HADS score across flooding impact terciles and plotted each city’s mean HADS and flood impact scores. We estimated Pearson correlation coefficients to measure the linear relationship between HADS and flood impact score using cutoffs at 0.2, 0.5, and 0.8 to define the relationship as weak, moderate, and strong, respectively.

We also used multilevel multivariate linear regression models to examine the linear association between HADS and flood impact scores. Additionally, we applied robust, multilevel Poisson regression models to assess the association between the proportion of moderate-to-severe anxiety and depressive symptoms and the burden of flooding in terciles. We reported results as prevalence ratios (PR) with 95% confidence intervals (95%CI). The multilevel structure accounted for individuals (Level 1) nested within macroregions of the state (Level 2), as flood exposure varied substantially across areas - some were heavily affected while others had minimal impact. This approach was essential given the contextual nature of the exposure: within each region, individuals shared similar levels of flood exposure. Ignoring this clustering could lead to collinearity among observations, biased standard errors, and potentially misleading effect estimates. A two-level model (individuals within regions) was therefore appropriate to disentangle individual and contextual effects and produce more accurate inferences.

We also compared the predicted and observed prevalence of moderate-to-severe symptoms of anxiety and depression at wave 6. Firstly, we fitted a multilevel Poisson regression model using data from waves 1 to 5, with moderate-to-severe symptoms of anxiety and depression (derived from the HADS) as the dependent variable. Independent variables included age, gender, race and ethnicity, marital status, schooling, income, and occupational status, all of which were collected consistently at each survey wave. Using this model, we employed the margins command in Stata software to generate predicted proportions of participants with moderate-to-severe symptoms at wave 6. Then, we ran an additional model including waves 1 to 6 and extracted the observed prevalence at wave 6. This procedure enabled us to assess whether the prevalence of moderate-to-severe anxiety and depressive symptoms during the flooding period (September 2024) deviated from predictions based on prior longitudinal patterns. All models accounted for clustering of individuals within macroregions of the state. Statistical significance was set at p < 0.05. Analyses were conducted using Stata/MP 14.2 (https://www.stata.com), and figures were generated in R version 4.3.3 (http://www.r-project.org).

## Results

We analyzed data from 2,494 adults with a mean age of 43.1 ± 15.1 years, most of whom were women (69.6%). Table 1 shows the sociodemographic characteristics of the participants, categorized by the burden of the 2024 flooding.

**Table 1:** Characteristics of the sample. PAMPA cohort, Rio Grande do Sul, Brazil (n = 2,494).

Characteristics	2024 flooding impact		
	Low (n = 1,138)	Mild (n = 616)	High (n = 740)
	n (%)	n (%)	n (%)
Gender			
Man	318 (28.0)	189 (30.7)	227 (30.7)
Woman	819 (72.0)	424 (68.9)	505 (68.3)
Nonbinary	0 (0.0)	2 (0.3)	7 (0.9)
Age [mean (SD)]	43.7 (15.3)	42.1 (15.1)	42.3 (14.3)
Race and ethnicity	1 (0.1)	2 (0.3)	1 (0.1)
Asian			
Black	42 (3.7)	19 (3.1)	19 (2.6)
Brazilian Indigenous	3 (0.3)	0 (0.0)	0 (0.0)
Mixed-race	74 (6.5)	54 (8.8)	67 (9.1)
White	1,018 (89.5)	539 (87.5)	649 (87.7)
Other	0 (0.0)	2 (0.3)	4 (0.5)
Schooling			
Middle school or less	6 (0.5)	9 (1.5)	15 (2.0)
High school	337 (29.6)	176 (28.6)	217 (29.3)
Higher education or graduate studies	795 (69.9)	431 (70.0)	508 (68.6)
Occupational status			
Not working	108 (9.5)	55 (8.9)	68 (9.2)
Working	850 (74.8)	457 (74.2)	557 (75.3)
Studying	55 (4.8)	39 (6.3)	39 (5.3)
Retired	107 (9.4)	53 (8.6)	58 (7.8)
Other	17 (1.5)	12 (1.9)	18 (2.4)
Conjugal situation			
Living alone	361 (31.7)	229 (37.2)	271 (36.6)
With partner	777 (68.3)	387 (62.8)	469 (63.4)
Home situation			
No need to leave home	1,077 (94.8)	542 (88.0)	473 (64.0)
Left home, not affected	55 (4.8)	49 (8.0)	115 (15.6)
Left home, partial losses	3 (0.3)	23 (3.7)	117 (15.8)
Left home, lost everything	1 (0.1)	2 (0.3)	34 (4.6)

SD: standard deviation.

Overall, 83.8% scored above 0 on the flood impact scale, indicating that four in five participants were affected to some extent. Participants experiencing a higher burden were more likely to identify as Mixed-race, have completed middle school or lower education level, and live alone. Among those reporting high flood impact, 36% had to leave their homes, with 15.8% experiencing partial losses and 4.6% reporting total losses. In contrast, 94.8% of those in the low-impact group did not need to leave their homes.

**Figure 1:**
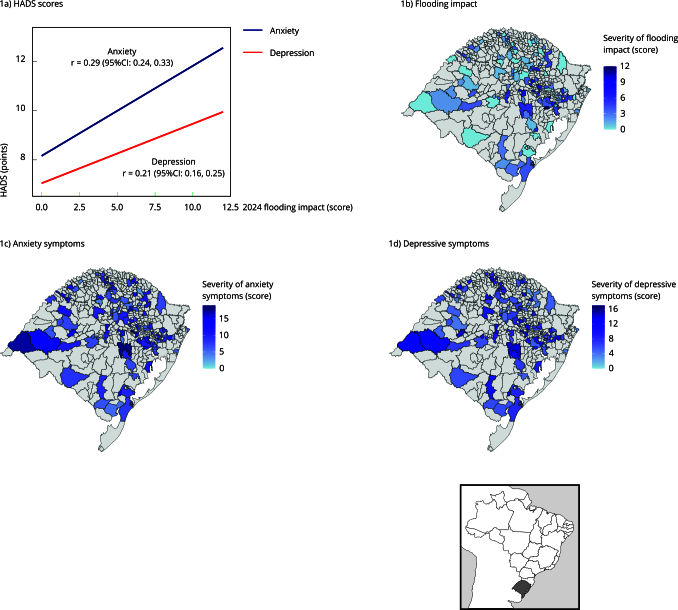
Linear association between *Hospital Anxiety and Depression Scale* (HADS) scores and flooding impact scores and mean scores by city. PAMPA cohort, Rio Grande do Sul, Brazil.

Figure 1 illustrates a moderate and statistically significant linear association between the impact of the 2024 flooding and anxiety symptoms (r = 0.29; 95%CI: 0.24, 0.33) and depression (r = 0.21; 95%CI: 0.16, 0.25). Regions with the highest mean scores on the HADS reported the greatest flooding burden, particularly in the central (Vales) and eastern (Metropolitana) regions of the state.

95%CI: 95% confidence interval. Note: Pearson’s correlation was used to assess the relationship between flooding impact and HADS scores.

**Figure 2:**
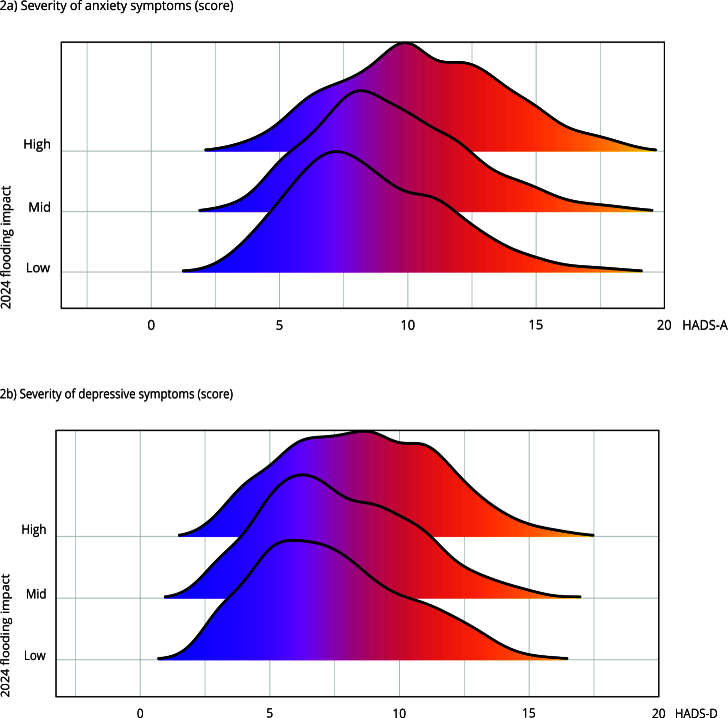
Distribution of *Hospital Anxiety and Depression Scale* (HADS) scores for anxiety and depression regarding the flooding impact terciles. PAMPA cohort, Rio Grande do Sul, Brazil.

Figure 2 illustrates the distribution of HADS scores for anxiety and depression regarding the flooding impact. Participants with the highest burden had significantly elevated HADS scores. Table 2 details that their anxiety and depression scores were, on average, 1.72 points (95%CI: 1.41, 2.03) and 0.88 points (95%CI: 0.59, 1.17) higher than those of participants with the lowest burden.

**Table 2:** Difference in *Hospital Anxiety and Depression Scale* (HADS) scores and the prevalence of moderate-to-severe symptoms according to flooding burden tertiles.

Flooding burden	Cases/N	Anxiety		Cases (%)	Depression	
		Coefficient (95%CI)	PR (95%CI)		Coefficient (95%CI)	PR (95%CI)
Low	622/1,052	1.00	1.00	483 (39.8)	1.00	1.00
Medium	435/586	0.77 (0.47, 1.08)	1.25 (1.07, 1.45)	293 (24.1)	0.31 (0.02, 0.59)	1.06 (0.85, 1.33)
High	567/698	1.72 (1.41, 2.03)	1.72 (1.49, 1.97)	437 (36.0)	0.88 (0.59, 1.17)	1.52 (1.25, 1.86)

95%CI: 95% confidence interval.

Note: flooding burden was described in score tercile. Coefficients were estimated using multilevel linear regression models, and prevalence ratios (PR) using multilevel Poisson regression models with robust variance, both accounting for clustering of individuals (Level 1) within regions (Level 2). All models included adjustments for age, gender, ethnicity, education, income, and occupational status.

Table 2 also highlights the likelihood of moderate-to-severe anxiety and depression symptoms based on the impact of the flooding. Most affected participants were 1.72 times (95%CI: 1.49, 1.97) and 1.52 times (95%CI: 1.25, 1.86) more likely to report moderate-to-severe anxiety and depressive symptoms, respectively, than those least affected.

Table 3 compares the expected versus actual proportions of moderate-to-severe anxiety and depression symptoms. For anxiety, the observed prevalence exceeded 36% of the expected proportion. Among participants with the highest flooding impact, this difference rose to 77%. For depression, the overall sample showed a 72% discrepancy between predicted and observed proportions, with a 129% difference for those experiencing the greatest burden. Notably, participants who were displaced due to the flooding had anxiety and depression proportions 80% and 89% higher, respectively, than the expected values.

**Table 3:** Expected versus actual proportion of moderate-to-severe anxiety and depressive symptoms after the 2024 Rio Grande do Sul flooding according to flooding burden tertiles. PAMPA cohort, Rio Grande do Sul, Brazil.

	Moderate-to-severe anxiety symptoms		Moderate-to-severe depressive symptoms	
	Expected	Actual	Expected	Actual
	Proportion (95%CI)	Proportion (95%CI)	Proportion (95%CI)	Proportion (95%CI)
Overall	17.7 (15.3, 20.2)	24.1 (20.7, 27.5)	8.5 (6.8, 10.2)	14.6 (11.8, 17.4)
2024 flooding impact				
Low	Not applicable	19.7 (15.5, 23.8)	Not applicable	12.8 (9.3, 16.3)
Medium	Not applicable	30.3 (22.8, 37.9)	Not applicable	15.7 (9.7, 21.8)
High	Not applicable	31.4 (22.3, 40.4)	Not applicable	19.5 (11.7, 27.2)
Displaced				
No	Not applicable	22.9 (21.8, 26.6)	Not applicable	14.4 (11.3, 17.4)
Yes	Not applicable	31.8 (21.8, 41.7)	Not applicable	16.1 (8.4, 23.8)

95%CI: 95% confidence interval.

Note: multilevel Poisson regression models with robust variance and 95%CI were used to compare predicted and observed proportions of moderate-to-severe anxiety and depressive symptoms. Predicted proportions were based on data from waves 1 to 5, while observed proportions included data from waves 1 to 6. The models accounted for the hierarchical structure of the data, with individuals (Level 1) nested within state regions (Level 2). All models were adjusted for age, gender, race and ethnicity, education, income, and occupational status.

## Discussion

Overall, four in five participants reported being affected by the flooding. Our results also revealed a significant rise in mental health symptoms after the flood, particularly among those who self-identified as mixed-race, had lower education levels (elementary school or less), and lived alone. Those most severely affected by the flood were 1.7 times and 1.5 times more likely to report moderate-to-severe anxiety symptoms depressive symptoms respectively. Notably, moderate-to-severe anxiety symptoms were 77% higher than expected. In comparison, depressive symptoms were 129% higher, highlighting the disproportionate mental health burden faced by these vulnerable groups in the aftermath of the disaster.

Recently, research on the mental health impacts of extreme weather events, particularly floods, has expanded ^7^. Floods cause significant environmental, economic, and health-related consequences ^1^ ^,^ ^2^. Studies have consistently linked flooding to increased symptoms of anxiety, depression, and post-traumatic stress ^18^ ^,^ ^19^ ^,^ ^20^ ^,^ ^21^. Reported prevalence rates vary widely, ranging from 2.2% to 84% for anxiety and 3.2% to 52.7% for depression, largely due to differences in study design, assessment tools, and follow-up duration ^13^ ^,^ ^22^. Even without the influence of climate change, these disorders are already a major global public health concern ^23^ ^,^ ^24^. However, the growing frequency and severity of extreme weather events may further intensify this burden ^1^ ^,^ ^2^.

Low- and middle-income countries are more affected by extreme weather events ^25^ ^,^ ^26^. Social inequalities and limited access to healthcare disproportionately impact vulnerable populations, such as women, children, low-income communities, individuals with lower educational attainment, those experiencing family instability, living alone, and those receiving inadequate social support ^18^ ^,^ ^20^ ^,^ ^27^ ^,^ ^28^. The inequality in exposure to environmental disasters, such as floods, reflects the socioeconomic disparities observed in our sample. Our findings show that individuals with lower educational attainment, those living alone, and mixed-race individual reported a higher burden of anxiety and depression symptoms. This unequal distribution of flood effects reinforces the environmental justice perspective, which argues that the impacts of climate change disproportionately affect vulnerable populations, exacerbating preexisting social inequalities ^29^.

Our findings also indicate a positive linear relationship between flood severity and mental health outcomes, which is consistent with previous research ^25^ ^,^ ^26^. Mental health issues following floods are often attributed to coexisting physical health issues, financial losses, and social or community disruption ^5^. The risk of psychological distress and fatalities increases with displacement and the breakdown of essential services ^11^. Moreover, strong connections to the environment and land-based cultural identity can intensify emotional distress in affected communities ^13^. Rather than the disaster itself, worsening economic conditions in its aftermath seem to drive posttraumatic symptoms, suggesting that chronic mental health challenges following disasters could be mitigated via economic support ^30^.

Psychological impacts of floods extend beyond the immediate event to the recovery period, which can last several months and be particularly challenging for groups with limited financial resources and social support networks. In our study, participants with lower educational attainment and those living alone were at greater risk of anxiety and depression symptoms, potentially reflecting difficulties in rebuilding their lives after the flood. These findings underscore the importance of public policies that provide ongoing financial and psychological support to vulnerable populations affected by natural disasters. Furthermore, these long-term effects pose enduring social and welfare challenge, not only as a direct consequence of flooding but also due to persistent secondary stressors associated with the recovery of lives, properties, and relationships ^18^.

This is the first study to examine the impact of flooding on individuals’ lives and depressive and anxiety symptoms among residents of Rio Grande do Sul. This study has several strengths. There were adults from multiple regions across the state, enhancing the generalizability of the findings. The prospective design also enabled predictive analyses, and multivariable models enabled adjustment for a range of potential confounding factors, providing a robust assessment of the associations examined. Nevertheless, the study also has some limitations. Data on flood impact and mental health symptoms were collected by a self-reported online questionnaire, which may have introduced selection bias; individuals most severely affected might not have had internet access and, therefore, could have been underrepresented, potentially leading to an underestimation of the effect sizes. Future research using longer follow-ups is essential to better understand mental health trajectories and the long-term impacts of flooding. Therefore, caution is advised when interpreting these findings.

## Conclusion

In conclusion, this study highlighted that more than 80% of individuals had their lives impacted by the 2024 flood in Rio Grande do Sul. Those who reported a higher burden from the flood demonstrated an increased likelihood of experiencing anxiety and depressive symptoms, particularly among less privileged individuals. Participants experiencing greater burden reported higher rates of moderate-to-severe symptoms. To mitigate the mental health impact, the government should develop comprehensive economic and mental health support systems as part of prevention, preparedness, response, and recovery plans, with a focus on the most vulnerable populations.

## Data Availability

The research data are available upon request to the corresponding author.

## Material Suplementar

Material Suplementar
