# Supplementary material for: Depressive and anxiety symptoms after the 2024 flooding in Rio Grande do Sul, Brazil: findings of the PAMPA cohort
Source: Cad Saude Publica. 2026 Feb 16;42:e00096225. doi: 10.1590/0102-311XEN096225 (PMC12919920; doi:10.1590/0102-311XEN096225)
Supplement: Material Suplementar [file 1678-4464-csp-42-EN096225-s.pdf]

SUPPLEMENTARY MATERIAL

AGORA VAMOS CONVERSAR SOBRE AS ENCHENTES NO RIO GRANDE DO SUL		
1	Você precisou sair de casa devido às enchentes?	○ Sim<br>○ Não
2	De que forma você foi afetado?	○ Tive que sair de casa, mas minha moradia não foi atingida diretamente pelas águas da enchente e não tive perdas materiais<br>○ Tive que sair de casa, perdi parcialmente minha moradia e bens materiais que foram atingidos pelas águas da enchente<br>○ Tive que sair de casa, perdi totalmente minha moradia e bens materiais que foram atingidos pelas águas da enchente
3	Você precisou sair da sua moradia com urgência?	○ Sim<br>○ Não
4	Você precisou de resgate?	○ Sim<br>○ Não
5	Você foi acolhido:	○ Na casa de outras pessoas<br>○ Em um abrigo público
6	Você ainda está morando em algum lugar diferente da sua casa por causa da enchente?	○ Sim<br>○ Não
7	Tive perdas materiais	○ Sim<br>○ Não
8	Fiquei sem água	○ Sim<br>○ Não
9	Fiquei sem luz	○ Sim<br>○ Não
10	Fiquei sem acesso à internet	○ Sim<br>○ Não
11	Fiquei sem suprimentos básicos (água potável e/ou alimentos)	○ Sim<br>○ Não
12	Tive contato com a água das enchentes	○ Sim<br>○ Não
13	Você contraiu alguma doença associada à enchente (leptospirose, diarreia, problemas de pele)?	○ Sim<br>○ Não
14	Perdi animais de estimação	○ Sim<br>○ Não
15	Meu estabelecimento de trabalho foi atingido e/ou fiquei impossibilitado de exercer meu trabalho	○ Sim<br>○ Não
16	Fui afetado pelo fechamento de aeroportos e/ou rodovias	○ Sim<br>○ Não
17	Fui afetado pelo fechamento de estradas e pela interrupção do funcionamento dos meios de transporte locais	○ Sim<br>○ Não
18	Fiquei impossibilitado de realizar ou dar continuidade ao meu tratamento de saúde pela interrupção do serviço e/ou impossibilidade de chegar até o local	○ Sim<br>○ Não
19	Como você avalia seu estado de saúde após esses eventos climáticos	○ Piorou minha saúde<br>○ Manteve-se a mesma<br>○ Melhorou minha saúde
20	Você recebeu algum auxílio financeiro devido à enchente?	○ Não<br>○ Sim, Auxílio Reconstrução (Governo Federal)<br>○ Sim, Auxílio A Casa é Sua – calamidades, Volta por Cima (Governo Estadual)<br>○ Sim, auxílios municipais
21	Como você considerava a qualidade da sua alimentação ANTES da enchente:<br><br>Boa = consumo PREDOMINANTE e regular de alimentos in natura ou minimamente processados, como frutas, legumes, verduras, raízes, grãos, sucos de frutas sem adição de substâncias, carnes, ovos, leite.<br><br>Ruim = consumo PREDOMINANTE e regular de alimentos	○ Boa<br>○ Ruim

	ultraprocessados, como refrigerantes, salgadinhos de pacote, chocolate, sorvete, pó para refrescos, pães de forma, macarrão instantâneo, guloseimas, biscoitos, misturas para bolo, cereais matinais, bebidas saborizadas, molhos prontos, produtos congelados para aquecer e outros produtos instantâneos.	
22	Você acha que a qualidade da sua alimentação DURANTE a enchente:	○ Piorou<br>○ Manteve-se a mesma<br>○ Melhorou
23	Você acha que a qualidade da sua alimentação após a enchente (comparada ao período anterior à enchente)	○ Piorou<br>○ Manteve-se a mesma<br>○ Melhorou
